# Nonculture Diagnostic Tests for Enteric Diseases

**DOI:** 10.3201/eid1803.111914

**Published:** 2012-03

**Authors:** Timothy F. Jones, Peter Gerner-Smidt

**Affiliations:** Tennessee Department of Health, Nashville, Tennessee, USA (T.F. Jones);; Centers for Disease Control and Prevention, Atlanta, Georgia, USA (P. Gerner-Smidt)

**Keywords:** acute gastroenteritis, diagnostic testing, nonculture methods, bacteria, enteric diseases, Shiga toxin–producing Escherichia coli, Campylobacter

The diagnosis of acute gastroenteritis (AGE) has traditionally been based on culture results of feces from patients with diarrhea. Virtually everything we know about disease and the epidemiology of enteric pathogens, such as *Salmonella* spp., Shiga toxin–producing *Escherichia coli* (STEC), e.g., O157, and *Campylobacter* spp., has been generated from the study of patients with culture-confirmed infections. However, this pattern may be changing because AGE diagnostics are moving away from culture toward rapid nonculture methods. These infections are mainly foodborne and therefore preventable, and it is of paramount importance that public health surveillance for these infections is consistent and reliable.

Reports by Stigi et al. (*1*) and M’ikanatha et al. (*2*) in this issue of the journal on changing laboratory practices for the testing of stool specimens illustrate this point, and raise serious issues for clinicians and the public health community. These 2 studies examined different pathogens, but both highlight the need to adapt policies and practices to keep up with rapid technical changes in the clinical laboratory world.

As Stigi et al. demonstrate, laboratory practices of testing for STEC are changing dramatically (*1*). In their study during 2005–2010, the number of laboratories performing antigen tests for Shiga toxin increased 8-fold. Although more than half of fecal specimens tested in Washington State, USA, were assayed for Shiga toxin, it is worrisome that 13% were tested for toxin alone, without concomitant culture. M’ikanatha et al. similarly reported that in Pennsylvania, USA, the number of laboratories submitting STEC antigen–positive culture broths more than doubled from 2009 through 2011, which indicated a major change in diagnostic practice (*2*).

For clinical purposes, it is generally sufficient to know that an STEC is present because management of an individual case is seldom dependent on additional subtyping. An unfortunate consequence of the increasing use of nonculture diagnostic tests for AGE is that they do not provide isolates for additional testing by public health laboratories. Public health has traditionally relied upon cultured organisms for further characterization, including subtyping for epidemiologic purposes. For this reason, in 2009 the Centers for Disease Control and Prevention published guidelines for the diagnosis of STEC by clinical laboratories (*3*). These guidelines recommend simultaneous culture for STEC O157 and for detection of Shiga toxin and forwarding of isolates or Shiga toxin–positive broths to public health laboratories for further characterization.

The study by M’ikanatha et al. also examined laboratory practices regarding identification of *Campylobacter* spp (*2*). In their study, use of nonculture diagnostic tests was substantial: in 17% of laboratories that used commercial fecal antigen tests for detecting *Campylobacter* spp.; all but one used only the antigen assay. As with STEC, such practices result in no isolates being available for additional testing by public health laboratories. For *Campylobacter* spp., this approach may be of somewhat less concern because in many states this pathogen is not reportable, molecular subtyping is not routinely performed, and outbreaks are relatively rare. However, it is emblematic of the overall trend away from culturing in commercial laboratories.

With the inexorable shift away from traditional laboratory methods in the clinical world, public health laboratories will increasingly face the challenge of having to develop the capacity to routinely isolate, characterize, and subtype pathogens from clinical specimens to gather the information on which epidemiologists have become so dependent. For example, if clinical laboratories diagnose STEC without culture results, outbreak detection will be more difficult. Molecular subtyping is now relied on heavily to identify small clusters of potentially related infections before the number of cases is epidemiologically evident. In many respects, loss of this resource would be a step 2 decades backward to the pre–pulsed-field gel electrophoresis era.

In addition, implementation of nonculture diagnostic methods introduces a bias in surveillance of AGE. For example, public health surveillance for STEC has traditionally focused on *E. coli* O157, and culture confirmation is still required for counting these cases in national data (*4*). In 2000, non-O157 STEC became nationally reportable, but numbers remained low until toxin testing became widely available. The recent rapid increase in reported non-O157 STEC is not unique to the studies reported in this issue (*5**–**7*), and as those cases have increased, the number of reported *E. coli* O157 cases has decreased. It is likely that a substantial proportion of STECs identified only by antigen testing are O157 (50% in 1 study) (*5*). Therefore, it is necessary to take changing diagnostic methods into account if trends in AGE are to be assessed accurately.

The sensitivity, specificity, and associated positive and negative predictive values of antigen tests for enteric pathogens also differ from those of culture, which makes it difficult to include the results of such tests as part of the definition of reportable diseases. Although such concerns are valid, policies must be developed that take into account changes in laboratory practices when evaluating trends in these pathogens. Scientific rigor is needed, but one must remember that clinicians respond to test results that they receive, and they trust that commercially performed tests are reliable. Regardless of how accurate is the testing method, the patient is being notified and treated on the basis of these test results, and public health officials must respond promptly on the basis of the information available. Although it is reasonable to keep data on cases of diseases diagnosed by using culture and nonculture methods separate, these data should be monitored so as not to lose essential information regarding the incidence of these diseases.

The repertoire of methods and targets for fecal testing is rapidly expanding. Molecular diagnostics are increasing; improvements include multiplex and quantitative PCR, fluorescence in situ hybridization, and metagenomic analyses (*8**–**10*). It is likely that many isolate-based methods for serotyping, pulsed-field gel electrophoresis, and antimicrobial drug testing will need to transition to sequence-based techniques to remain epidemiologically useful.

If these challenges are to be overcome, several issues must be addressed. Decisions about implementation of new methods in clinical laboratories are often based mostly on cost and ease of use, whereas parameters such as their sensitivity, specificity, and relevance to public health surveillance are less likely to be emphasized. However, all these aspects should be considered carefully before new diagnostic methods are implemented in clinical laboratories. If this does not happen, surveillance for foodborne AGE is likely to become unreliable and unsuitable for guiding public health actions in the future.
